# Tuning of structural, optical band gap, and electrical properties of room-temperature-grown epitaxial thin films through the Fe_2_O_3_:NiO ratio

**DOI:** 10.1038/s41598-019-41049-9

**Published:** 2019-03-13

**Authors:** Okkyun Seo, Akhil Tayal, Jaemyung Kim, Chulho Song, Yanna Chen, Satoshi Hiroi, Yoshio Katsuya, Toshiaki Ina, Osami Sakata, Yuki Ikeya, Shiori Takano, Akifumi Matsuda, Mamoru Yoshimoto

**Affiliations:** 1 Synchrotron X-ray Group, Research Center for Advanced Measurement and Characterization, National Institute for Materials Science (NIMS), Kouto, Sayo, Hyogo, 679-5148 Japan; 2 Synchrotron X-ray Station at SPring-8, Research Network and Facility Services Division, NIMS, Kouto, Sayo, Hyogo, 679-5148 Japan; 3 Research & Utilization Division, Japan Synchrotron Radiation Research Institute (JASRI), Kouto, Sayo, Hyogo, 679-5148 Japan; 4 Department of Materials Science and Engineering, School of Materials and Chemical Technology, Tokyo Institute of Technology, 4259-J3-16, Nagatsuta, Midori, Yokohama, 226-8502 Japan

## Abstract

We have investigated the structural, optical band gap, and electrical properties of (Fe_2_O_3_)_0.5*x*_:(NiO)_1 − 0.5*x*_ (*x* = 0.3, 0.4, 0.5, 0.6 and 0.7) epitaxial thin films grown on an atomically smooth substrate at room temperature. With increasing Fe_2_O_3_ content, the rock-salt structure of the thin films transformed to a spinel structure above *x* = 0.6. In terms of the local structure, the increased ratio of Fe^2+^ ions to Fe^3+^ ions indicates that the octahedral sites of FeO were continuously transformed into distorted octahedral and tetrahedral sites. On the other hand, the NiO matrix was not affected by the local structure change. Chemical composition of Fe_2_O_3_:NiO affected the crystal structure, the electrical conductivity and the optical band gap of direct transition (3.35 to 2.99 eV).

## Introduction

Nickel oxide (NiO) is an important semiconductor with a rock-salt (NaCl type) crystal structure^1,2^. NiO is antiferromagnetic with a Néel temperature of 523 K^3,4^. Although a NiO thin film with perfect stoichiometry is an insulator with a resistivity of approximately 10^13^ Ωcm, it easily contains intrinsic Ni vacancies and thus behaves as a *p*-*type* semiconductor with a direct band gap^5^. Moreover, its band gap is 3.6–4.0 eV, and its optical transparency with thermal and chemical stabilities in air is excellent^6^. Therefore, it is an attractive material for multifunctional use in devices such as transparent conducting oxides, organic solar cells, field effect transistors, ultraviolet (UV) photodetectors, UV light emitting diodes, and gas sensors^7–11^.

NiO thin films can be doped with elements such as Li, Mg, and Fe to tune the band gap and modify the electrical conductivity, optical band gap and magnetic properties. Doping with Mg leads to widening of the band gap, whereas doping with Li or Fe leads to narrowing of the band gap^3,6,12–14^. Further, modifying the doping material and content for NiO thin films can provide new electrical and optical properties.

A Fe-doped NiO thin film with the rock-salt structure can exhibit ferromagnetism, and the magnetization can be tuned by adjusting the doping concentration^15^. It is a promising candidate for *p*-*type* diluted metal semiconductors in semiconductor spintronics. In 2014, Zhang *et al*. reported that a strain between the substrate and thin films affected the superexchange interaction with Ni and Fe ions^3^. The antiferromagnetic exchange interaction of Fe-doped NiO thin films is enhanced by the lattice strain. Highly Fe-doped NiO thin films can be transformed into NiFe_2_O_4_ thin films with spinel structure. Spinel ferrite materials exhibit room temperature (RT) ferrimagnetism and possess a wide band gap^16–18^. The magnetic and electrical properties are dependent on the crystal quality and orientation^19^. Therefore, the crystal structure and local structure of Fe-doped NiO and NiFe_2_O_4_ thin films affect the magnetic, optical, and conductivity properties. Although many studies on the magnetic properties of Fe-doped NiO and NiFe_2_O_4_ thin films have been performed; there is a lack of research on the optical properties, electrical conductivity, and crystal structure of Fe_2_O_3_:NiO mixture thin films^3,15,19–21^.

*α*-Fe_2_O_3_ is a promising *n*-*type* semiconductor material for photocatalytic and photoelectrochemical applications because of its broad solar spectrum resulting from its wide band gap of 1.9–2.2 eV (*λ* = 650–560 nm) and its high chemical stability^22,23^. Although *α*-Fe_2_O_3_ is an insulator with an electrical resistivity of 10^14^ Ωcm in its stoichiometric form, the electrical resistivity can be reduced to 4–75 Ωcm by substitutional doping such as Ni, Cu, Mg, and Ti^23–25^.

Herein, a periodic step array of the thin films with Fe_2_O_3_:NiO ratio under compressive strain were grown on an atomically stepped sapphire (0001) substrate at RT to understand the effect of doping on the crystal structure, local structure, and optical and electrical properties. The atomic step array formation and surface morphology of the thin films were investigated using atomic force microscopy (AFM). The lattice constants, strain, epitaxial relation, and crystalline domain were evaluated using synchrotron X-ray diffraction (XRD). The local atomic structure and local charge of the thin films were modeled and evaluated using X-ray absorption fine structure (XAFS) analysis. From the electronic and optical measurements, we were able to explain how the structural disorder, distorted octahedral site, and local structure tuning affect the electrical conductivity in (Fe_2_O_3_)_0.5*x*_:(NiO)_1−0.5*x*_ thin films.

## Results and Discussion

**Figure 1:**
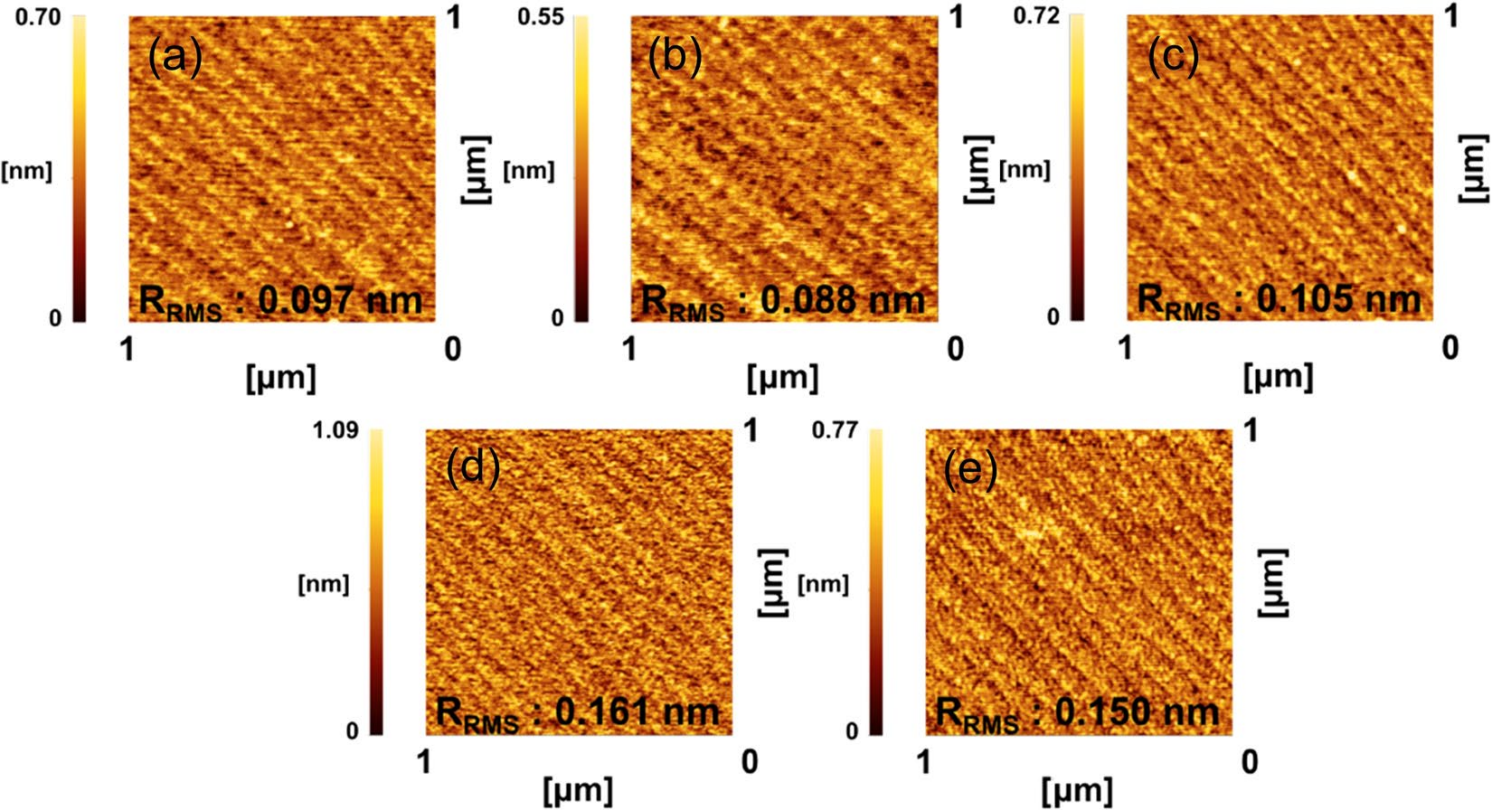
AFM images of as-grown (Fe_2_O_3_)_0.5*x*_:(NiO)_1−0.5*x*_ thin films on the sapphire substrate as a function of the Fe_2_O_3_ content, which are *x* = (**a**) 0.3, (**b**) 0.4, (**c**) 0.5, (**d**) 0.6, and (**e**) 0.7 respectively.

Figure 1 shows the morphology and roughness of the thin films on the sapphire substrate obtained from AFM measurements (1 × 1 *μ*m^2^) in tapping mode. Periodic step patterns (step height of 0.2 nm) were fabricated on the sapphire substrate, as shown in Fig. S1(a). Periodic step patterns of the film surface on the sapphire substrate were also formed, indicating that the 111-oriented thin films were grown along the substrate atomic steps. In other words, the surfaces of the thin films were quite flat surfaces, reflecting the atomic step and terrace morphology of the substrates. The roughnesses of the thin films with *x* = 0.3, 0.4, 0.5, 0.6, and 0.7 were 0.097, 0.088, 0.105, 0.161, and 0.150 nm, respectively. With increasing Fe_2_O_3_ content, the surface roughness tended to increase.

**Figure 2:**
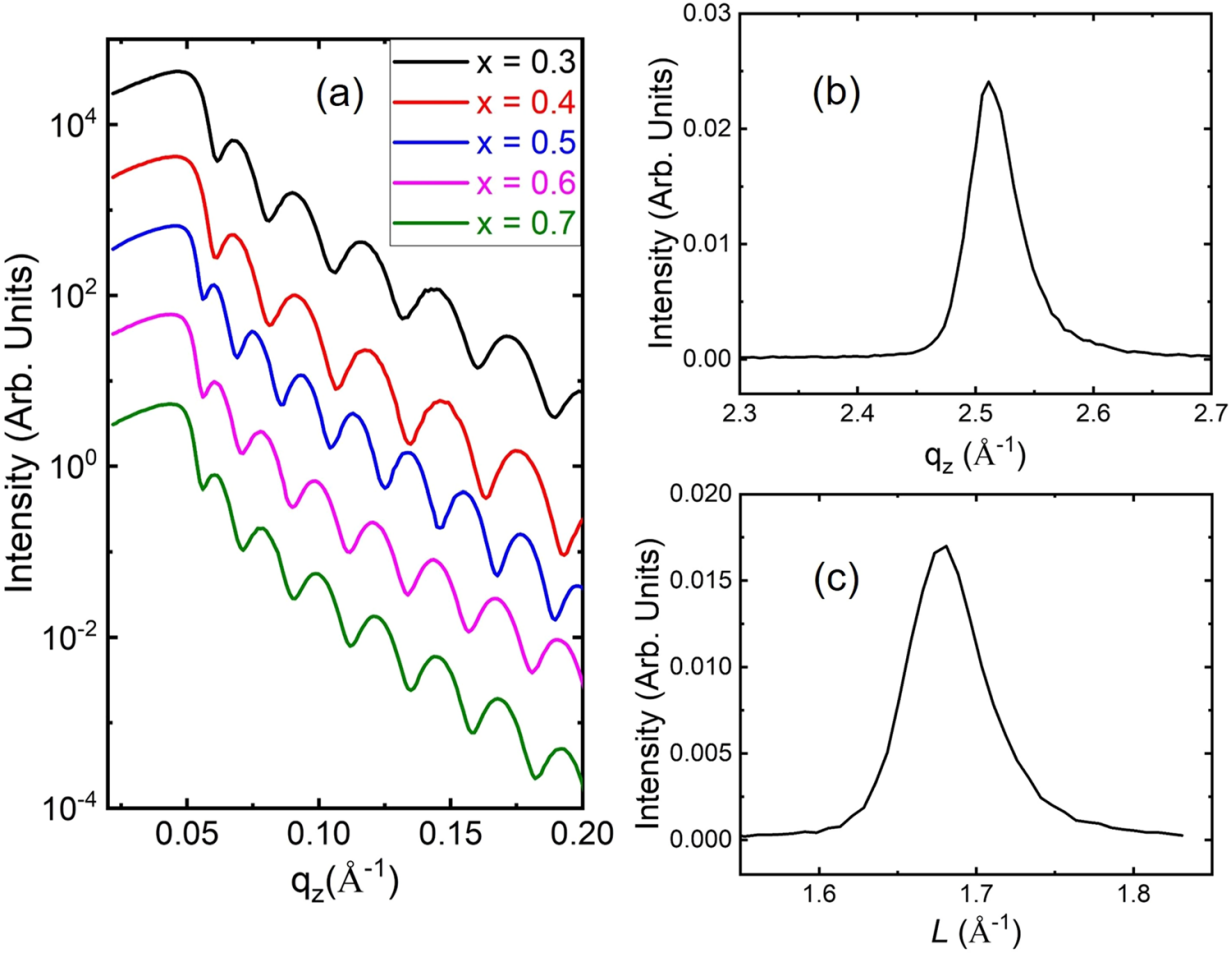
(**a**) X-ray reflectivity profiles of (Fe_2_O_3_)_0.5*x*_:(NiO)_1−0.5*x*_ thin films. (*x* = 0.3, black; 0.4, red; 0.5, blue; 0.6, pink; 0.7, olive) XRD profiles of thin film with *x* = 0.3 along (**b**) the substrate normal direction near the 111 Bragg reflection and (**c**) the off-specular 200 Bragg reflection.

Figure 2(a) presents the X-ray reflectivity results of the thin films as a function of the Fe_2_O_3_ content. The thickness *t* of the thin films evaluated from the reflectivity fringes Δ*q* for Fe_2_O_3_ contents *x* = 0.3, 0.4, 0.5, 0.6, and 0.7, for which the thickness was evaluated using the equation *t* = 2*π*/Δ*q*, were 23.2, 23.0, 31.8, 28.5, and 28.3 nm, respectively. The critical angles of the X-ray reflectivity results decreased with increasing Fe_2_O_3_ content, indicating that the atomic number density decreased. In other words, the porosity of the surface and ratio of Fe to O atoms increased. An XRD profile of the thin film with *x* = 0.3 along the substrate normal direction is presented in Fig. 2(b). The XRD peak near *q*_*z*_ = 2.498 Å^−1^ matched with the 111 Bragg reflection of the thin film with *x* = 0.3, which indicates a lattice constant along the normal direction of 4.3272 ± 0.0003 Å. The crystalline domain size along the out-of-plane direction evaluated from the half-width at half-maximum (HWHM) of the 111 Bragg peak, was approximately 13.2 nm. Figure 2(c) shows the off-specular 200 Bragg reflection of the thin film with *x* = 0.3. The thin film was epitaxially grown on a sapphire substrate with 6-fold symmetry (Fig. S2(c)). The lattice constant along the off-specular direction of the thin film with *x* = 0.3 was 4.2063 ± 0.0008 Å. This result indicates that the thin film grown on the sapphire 0001 substrate was in compressive strain with the rock-salt structure. The correlation length of the thin film with *x* = 0.3 along the off-specular direction estimated from the HWHM of the 200 Bragg reflection, was approximately 11.0 nm. Although the thin films were grown at RT, they exhibited relatively good crystallinity and high crystalline orientation along the substrate.

**Figure 3:**
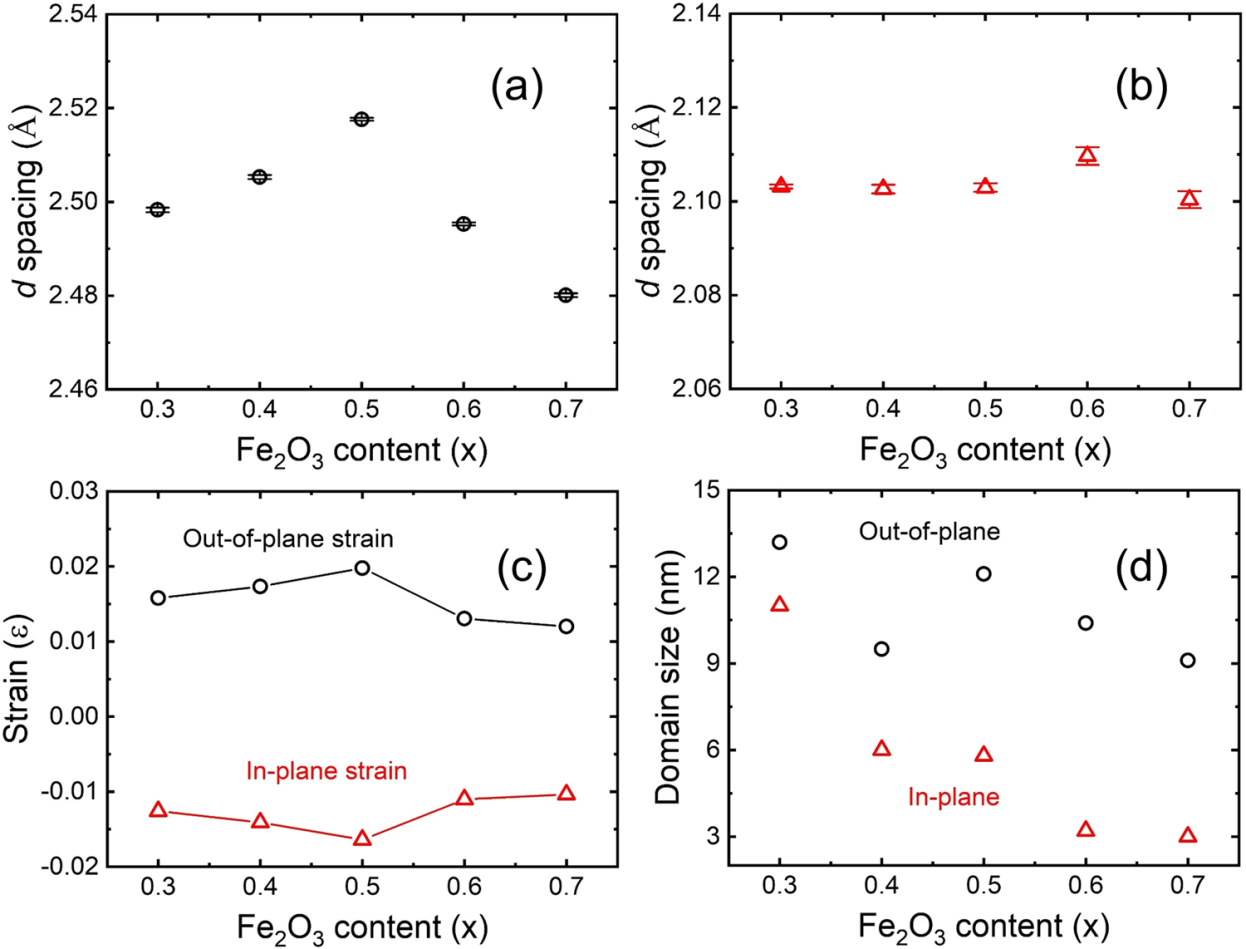
*d* spacing of the thin films along the (**a**) out-of-plane and (**b**) in-plane direction as a function of Fe_2_O_3_ content. (**c**) Out-of-plane strain and in-plane strain estimated from lattice constant. (**d**) Crystalline domain size of the thin films estimated from peak width of XRD results.

Figure 3(a,b) present the *d* spacings of the thin films as a function of the Fe_2_O_3_ content along the out-of-plane and in-plane directions. The *d* spacings of the thin films with Fe_2_O_3_ contents of 0.3, 0.4, 0.5, 0.6, and 0.7 at along the out-of-plane direction were 2.4983 ± 0.0005 Å, 2.5053 ± 0.0004 Å, 2.5176 ± 0.0003 Å, 2.4953 ± 0.0003 Å, and 2.4801 ± 0.0004 Å, as estimated from the XRD results in Fig. S2(a). The *d* spacings of the thin films along the in-plane direction were 2.1032 ± 0.0004 Å, 2.1026 ± 0.0009 Å, 2.1029 ± 0.0009 Å, 2.1097 ± 0.0019 Å, and 2.1004 ± 0.0019 Å, as evaluated from XRD results in Fig. S2(b). The lattice constants of bulk NiO and FeO are 4.177 Å and 4.295 Å, respectively^26,27^. The *d* spacings should have increased with the addition of Fe_2_O_3_; however, the *d* spacings tended to decrease at *x* = 0.6 and 0.7. Thus, the lattice constants of the thin films did not follow Vegard’s law^28^. This finding indicates that the crystal structure was changed with the addition of Fe_2_O_3_. At Fe_2_O_3_ contents of *x* = 0.3, 0.4, and 0.5, the thin films possessed the rock-salt crystal structure. However, the crystal structure of the thin films with *x* = 0.6 and 0.7 was a spinel structure, for which the lattice constant of bulk NiFe_2_O_4_ is 8.340 Å^19,20^. Therefore, the crystal structure transformation from the rock-salt to spinel type proceeds at higher Fe_2_O_3_ contents. Detailed results of the local structure (oxygen valence) will be discussed in the XAFS section. Using the lattice constants of the out-of-plane and in-plane directions, the in-plane strain of the thin films was evaluated^28–30^. The in-plane strain of the thin films is defined as^30^

1 $${\varepsilon }_{\parallel }=\frac{-\gamma }{\gamma +\frac{1+\omega }{1-\omega }}$$

where *ω* is the Poisson’s ratio and *γ* is the amount of tetragonal distortion, *γ* ≡ $\frac{{a}_{\perp }-{a}_{\parallel }}{{a}_{\parallel }}$. The *γ* values were estimated from the lattice constants, as shown in Fig. S3. *γ* of the thin film with *x* = 0.5 was higher than that of the other thin films, indicating that a higher content of Fe_2_O_3_ is related to tetragonal distortion before the phase transformation. After the phase transformation to the spinel structure, the tetragonal distortion was reduced. Poisson’s ratio of NiO and Fe_2_O_3_ are 0.399 and 0.240, respectively^31–33^. We applied the Poisson’s ratio of NiO and Fe_2_O_3_ using the chemical composition of the thin films. Figure 3(c) shows the out-of-plane and in-plane strain of the thin films. The strain relation of the thin films along the in-plane and out-of-plane directions is defined as

2 $${\varepsilon }_{\perp ,\parallel }=\frac{{a}_{\perp ,\parallel }-{a}_{0}}{{a}_{o}}$$

where *a*_0_ is the relaxed lattice constant of the thin films and ${\varepsilon }_{\perp ,\parallel }$ is the out-of-plane and in-plane strain. The relaxed lattice constant is represented by the lattice constant of strain-free thin films, which is not affected by the substrate. The degree of tetragonal distortion is related to the out-of-plane and in-plane strains.

Figure 3(d) shows the crystalline domain size of the thin films along the out-of-plane and in-plane directions. The crystalline domain size was reduced with increasing Fe_2_O_3_ content when the thin film thickness was considered. This finding indicates that the crystallinity of the thin films became poorer.

**Figure 4:**
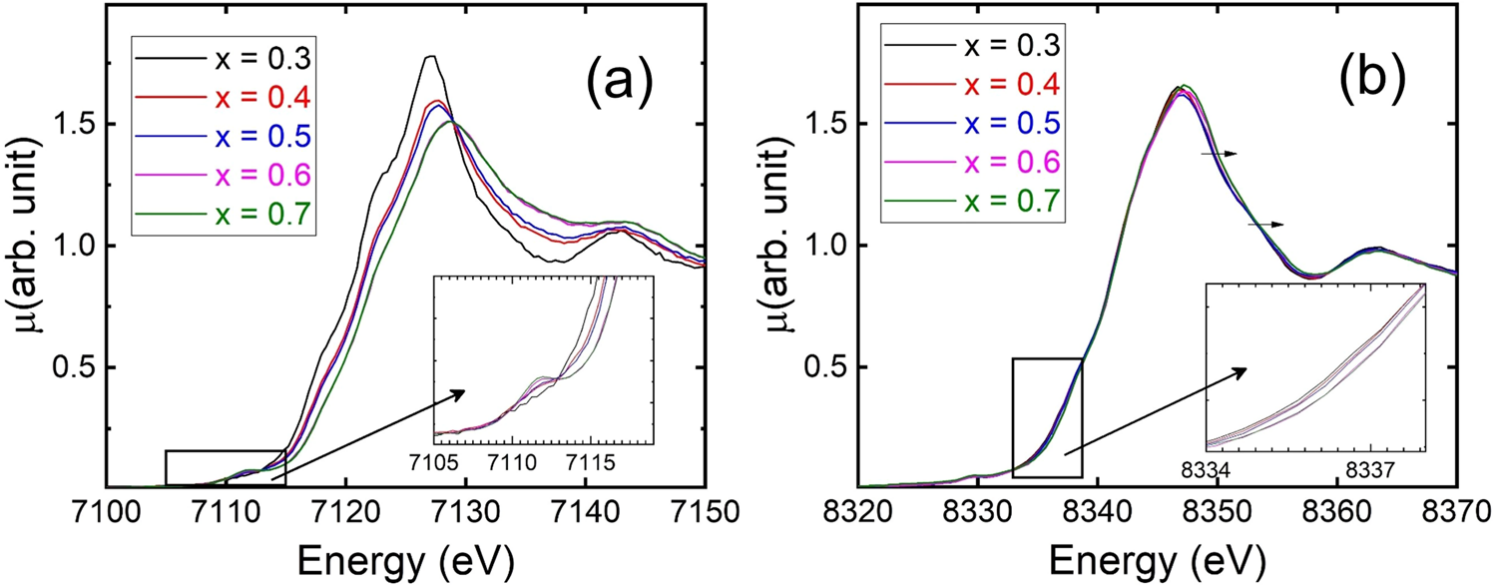
(**a**) XANES spectra at Fe *K*-edge as a function of Fe_2_O_3_ content. The inset shows the zoomed-in pre-edge region. (**b**) XANES spectra at Ni *K*-edge. The inset shows the zoomed-in region near 50% absorption, revealing the non-trivial difference in the edge position. The arrows drawn above the edge indicate shifting of the spectrum toward high energy with increasing Fe_2_O_3_ content.

Figure 4(a) presents the XANES profiles measured around the Fe *K*-edge with the inset showing the zoomed-in region near the pre-edge. The values of the edge position for all the samples were estimated at 50% absorption and were compared with the values obtained for FeO and Fe_2_O_3_, as shown in Table 1. The edge position with *x* = 0.3 was closer to that of the FeO reference sample, in which Fe has a +2 valence state. The edge positions were shifted to higher values and the pre-edge intensity increased with increasing Fe_2_O_3_ content, indicating an increase of the Fe valence. The observed pre-edge peak could arise either from an electric quadrupole transition or a dipole transition (1 *s* → 3 *d*). In general, the latter is forbidden because of the dipole selection rule; however, for a non-centrosymmetric environment, it is allowed because of the mixing of Fe 3 *d* with Fe 4 *p*/O 2 *p* orbitals^34^.

**Table 1:** Fe *K*-edge position of the thin films and reference samples.

Fe_2_O_3_ content (*x*)	Edge position	Reference sample	Edge position
0.3	7117.6 eV	FeO	7117.5 eV
0.4	7118.6 eV		
0.5	7118.8 eV		
0.6	7119.6 eV		
0.7	7119.6 eV	Fe_2_O	7121.6 eV

A value obtained at the 50% of absorption in the XANES spectra.

Wilke *et al*.^34^, investigated modification of the pre-edge features in a series of Fe compounds. It was observed that the centroid position of the pre-edge in Fe^2+^ compounds is generally 1.4 eV smaller than that of compounds bearing the Fe^3+^ state. Furthermore, the total integrated intensity of the pre-edge is observed to have the lowest value for compounds in which Fe has the most centro-symmetric environment. The blue-shift in the edge position with increasing Fe_2_O_3_ content indicates that the valence state of Fe is approaching +3. In addition, the increase in the pre-edge intensity indicates that the Fe local geometry undergoes a transformation from the symmetric octahedral (for *x* = 0.3) to tetrahedral/distorted-octahedral geometry with increasing Fe_2_O_3_ content. Figure 4(b) presents XANES profiles of the thin films at the Ni *K*-edge. Compared with the results obtained at the Fe *K*-edge, only a marginal shift toward higher energy was observed for the samples with *x* = 0.6 and 0.7. In addition, no pre-edge peak appeared, which signifies that the dominant Ni species in the film is composed of a NiO_6_ structural motif with a symmetric octahedral environment, as observed in the NiO compound.

**Figure 5:**
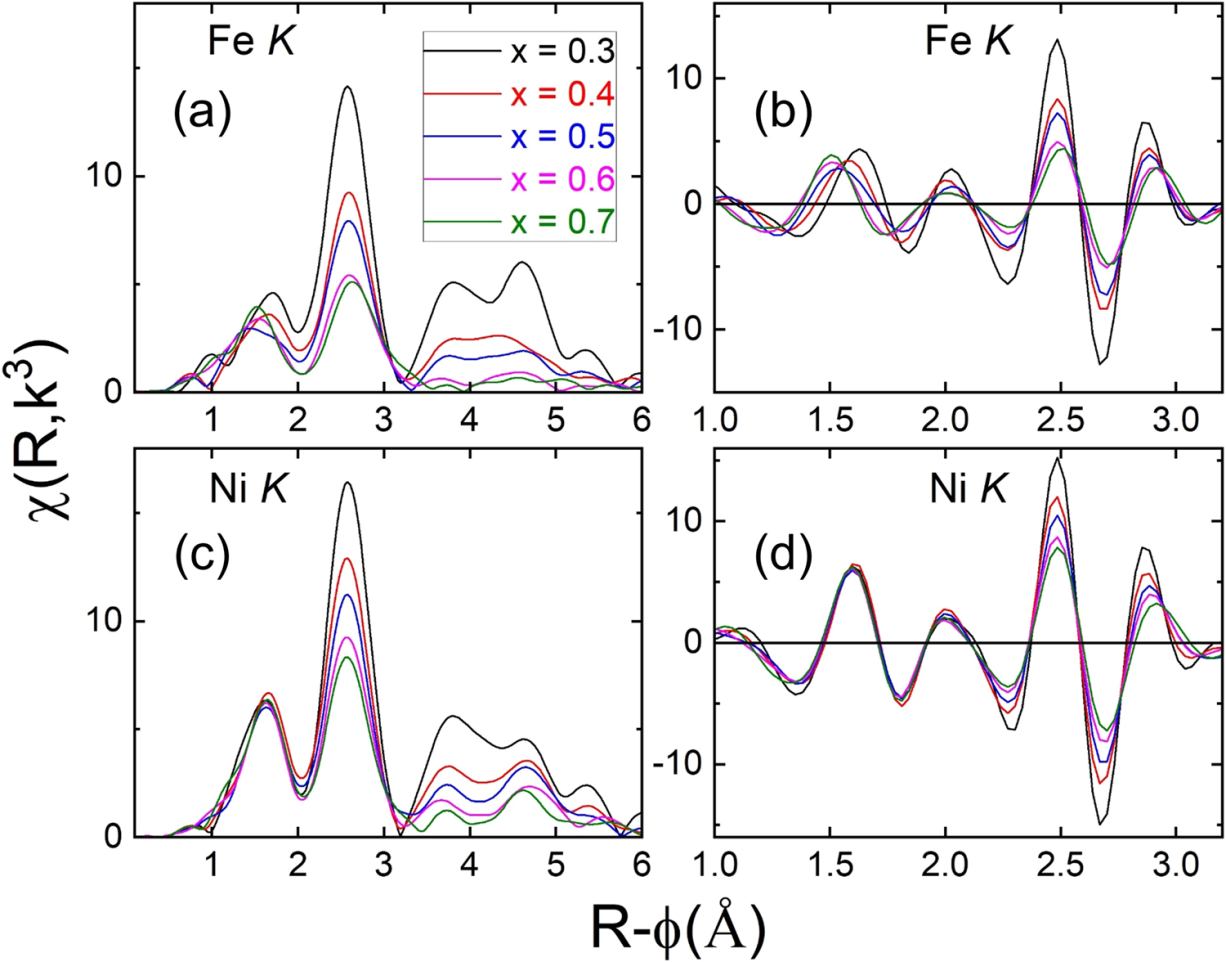
Fourier transform and real components of EXAFS results as a function of Fe_2_O_3_ content at Fe *K*-edge (**a**,**b**) and Ni *K*-edge (**c**,**d**), respectively.

To obtain further insight into the local geometry around the Fe and Ni atoms, the Fourier transform (FT) and real components of extended X-ray absorption fine structure (EXAFS) spectra measured at the Fe *K*-edge and Ni *K*-edge were investigated, as shown in Fig. 5. The two peaks observed in the FT spectra between 1–2 Å and 2–3.2 Å correspond to the first metal-oxide shell and second metal-metal shell, respectively, for both the edges. This variation in the Fe-O bond length correlates with the previously discussed XANES results, indicating that the valence state of Fe transformed from +2 to a mixture of (+2/+3) valence states with increasing Fe_2_O_3_ content. The increase in the Fe valence was concomitant with the formation of distorted Fe-O local geometry. The rise in the pre-edge intensity caused by the creation of tetrahedral/distorted-octahedral Fe sites supports the formation of shorter Fe-O bonds, similar to those observed in magnetite and hematite structures. In contrast, the FeO phase in which Fe is in a symmetric octahedral environment has a relatively longer Fe-O bond length.

Figure 5(c,d) present FT and real-component spectra measured at the Ni *K*-edge. The first shell region between 1–2 Å shows only a marginal variation in the FT peak position and real-component node position. This result indicates that the Ni-O bond length and symmetric octahedral environment around Ni were un-perturbed with increasing Fe_2_O_3_ content. However, the *χ*(R) amplitude showed distinct behavior for the spectra at the Fe *K*-edge and Ni *K*-edge. The *χ*(R) amplitude at the Fe *K*-edge was abruptly diminished in the region of higher-order shells (between 3–6 Å) for the samples with compositions above *x* = 0.6. Notably, in the FT spectra measured at the Ni K-edge, the features at 3.7, 4.7, and 5.6 Å, corresponding to higher shells, are still resolvable even for the sample with the highest Fe_2_O_3_ content. This result indicates that the local structure of Ni remained identical to that of NiO_6_. However, the abrupt drop in the high R features in the Fe *χ*(R) spectra could arise from the simultaneous variation in the local geometry and increasing structural disorder.

**Figure 6:**
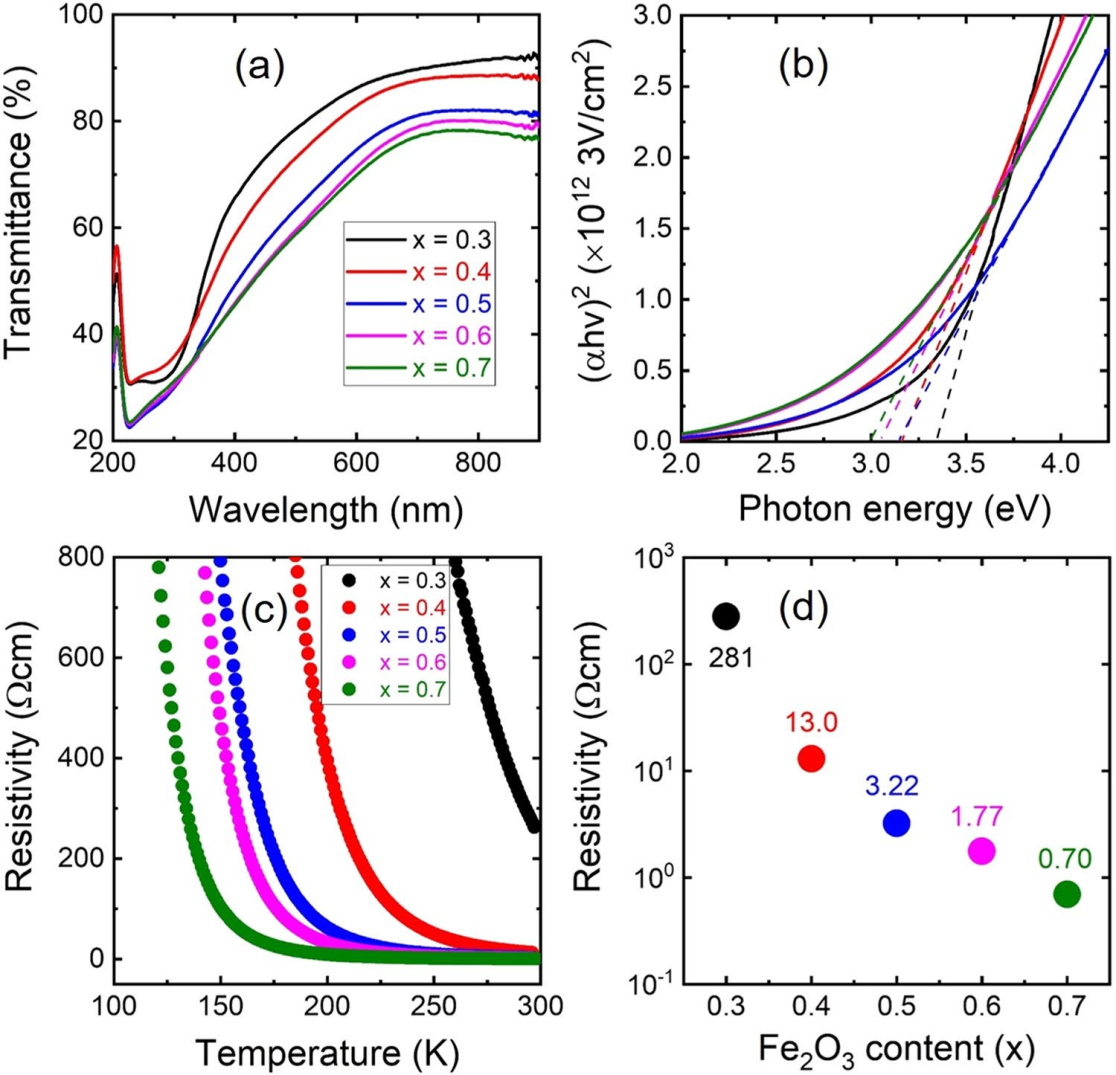
(**a**) Optical transmittance of the thin films in wavelength range of 200~900 nm. (**b**) Tauc’s plot of the thin films, with the data obtained from the optical transmittance spectra. The optical band gaps of direct transition for Fe_2_O_3_ contents *x* = 0.3, 0.4, 0.5, 0.6, and 0.7 were 3.35, 3.15, 3.14, 3.05, and 2.99 eV, respectively. (**c**) Electrical resistivity of the thin films as a function of temperature. (**d**) Electrical resistivity of the thin films at RT.

Figure 6(a) presents the transmittance spectra of the thin films as a function of the Fe_2_O_3_ content. The absorption edge of these films was shifted toward higher wavelength and their transmittance value was reduced with increasing Fe_2_O_3_ content. Figure 6(b) plots the optical band gaps of the thin films, evaluated using a Tauc’s plot using the relation between (*αhν*)^*n*^ and *hν* from the absorption edge determined from UV-VIS spectroscopy, where *α* is the optical absorption coefficient, *h* is Planck’s constant, *ν* is the photon frequency, and *n* = 2 for direct transition^35^. The optical band gap of direct transition was tuned from 3.35 to 2.99 eV with increasing Fe_2_O_3_ content. Because the optical band gap of *α*-Fe_2_O_3_ is 1.9–2.2 eV, the optical band gap of the thin films was shifted to narrower band gaps, as illustrated in Fig. 6(b)^22^. The optical band gaps of in-direct transition for n = 0.5 were 1.27 to 0.78 eV with increasing Fe_2_O_3_ content, as shown in Fig. S8.

To examine the electrical properties of the thin films, the temperature dependence of the resistivity is presented in Fig. 6(c). The resistivity continuously decreased in the temperature range between 100 K and 300 K, indicating the increased mobility of the charge carriers. The electrical resistivity at RT for *x* = 0.3, 0.4, 0.5, 0.6, and 0.7 was 281, 13.0, 3.22, 1.77, and 0.70 Ωcm, respectively, as illustrated in Fig. 6(d). This result indicates that higher Fe_2_O_3_ contents resulted in better electric conductivity, which is consistent with the decreased optical transmittance results. The formation of a small polaron (the self-trapped electron change of a particular ion) in the transition metal oxide can explain the carrier transport^25,36^. A self-trapped electron rarely occurs in FeO. However, Fe_2_O_3_ is a typical small polaron conductor, which results in strong interaction between charge carriers^36,37^. According to the EXAFS results, the FeO octahedral site transformed to a mixture of distorted-octahedral and tetrahedral sites with increasing Fe_2_O_3_ content, indicating that the bond length becomes shorter. Therefore, the polaron mobility is enhanced by the reduction of the hopping activation energy^25^. In addition, the use of an ionic oxide semiconductor with low crystallinity, a low degree of disorder, and a rough surface resulted in improved electronic conductivity^38^.

## Conclusion

Epitaxial (Fe_2_O_3_)_0.5*x*_:(NiO)_1−0.5*x*_ thin films were smoothly grown along the substrate step with rms roughnesses of 0.08~0.16 nm using PLD at RT. With increasing Fe_2_O_3_ content, the optical band gaps became narrower and were approximately 2.98–3.35 eV. Substitutional Fe doping, octahedral distortion, and the number of tetrahedral sites increased. As a result, the rock-salt structure transformed into a spinel structure with poorer crystallinity. The electrical resistivity of the thin films was reduced from 281 to 0.70 Ωcm, which was correlated to the substitutional doping, local structure, disordering, and crystal structure.

## Methods

### Sample preparation

The thin films were grown on atomically stepped single crystal sapphire (0001) substrates using pulsed laser deposition (PLD) at RT. To examine the doping effects of Fe ions on NiO thin films, sintered (Fe_2_O_3_)_0.5*x*_:(NiO)_1−0.5*x*_ with target chemical compositions were fabricated using a combination of NiO and Fe_2_O_3_ powder. First, ultra-smooth sapphire substrates with double-side polishing were fabricated using a thermal annealing process at 1273 K for 3 h in air^39,40^. The sapphire substrates were cleaned with acetone and ethanol in an ultrasonic bath. The thin film growth by PLD occurred in an ultra-high vacuum chamber (base pressure ~10^−6^ Pa) equipped with an *in situ* reflection high-energy electron diffraction (RHEED) observation system. A pulsed KrF excimer laser with a wavelength of 248 nm was used. The energy density, repetition rate, and pulse duration of the pulsed laser were 1.5 J/cm^2^, 5 Hz, and 20 ns, respectively. The partial oxygen pressure of the thin films during the sample growth was maintained at 10^−5^ Pa. The distance between the target and substrate was maintained at 5 cm. The surface morphology and roughness of the thin films were evaluated using AFM (Hitachi High-Tech Science, Nanocute, Japan) in tapping mode.

### X-ray diffraction

High-resolution synchrotron XRD measurements were performed at the BL15XU NIMS beamline at SPring-8 in JAPAN. The incident X-ray wavelength was fixed at 1 Å using a Si 111 monochromator with an undulator. The thicknesses of the thin films were evaluated from X-ray reflectivity profiles of the thickness fringes. To investigate the tetragonal distortion, strain, and crystalline domain size, we investigated the 111 Bragg reflection along the substrate normal direction as well as the 002 Bragg reflection along the off-specular direction. The tetragonal distortion and strain of the thin films were determined by measuring the 111 and 002 peaks. The [111] direction of the thin films was grown in the [0001] direction of sapphire substrate; the in-plane [11$\bar{2}$] direction was parallel to the [11$\bar{2}$0] direction of the sapphire substrate.

### X-ray absorption fine structure

XAFS measurements were performed at BL01B1 beamline at SPring-8. The incident X-ray energy was tuned using a Si 111 double-crystal monochromator. EXAFS results were obtained near the Ni and Fe *K*-edge using a Ge solid-state detector at 90° in the horizontal plane to the incident X-ray beam. The energy of the obtained spectrum ranged from 200 to 800 eV relative to the Fe and Ni *K*-edge energies. The X-ray energies were calibrated using metal and metal oxide reference samples.

### Optical and electrical properties

The optical transmittance and band gap of the thin films were measured using UV-vis spectroscopy (JASCO, V-550) in the optical region from 200 to 900 nm. The optical transmittance was evaluated by subtracting the double-side polishing substrate measurement from that of the film grown on the substrate. The electrical conductivity of the thin films was estimated by examining the resistivity measurement as a function of temperature.

## Supplementary information

Supplementary information
