# Supplementary material for: Tuning of structural, optical band gap, and electrical properties of room-temperature-grown epitaxial thin films through the Fe2O3:NiO ratio
Source: Sci Rep. 2019 Mar 13;9:4304. doi: 10.1038/s41598-019-41049-9 (PMC6416339; doi:10.1038/s41598-019-41049-9)
Supplement: Supplementary file 1 — Supplementary information [file 41598_2019_41049_MOESM1_ESM.pdf]

Supplementary information for

Tuning of structural, optical band gap, and electrical properties of room-temperature-grown epitaxial thin films through the Fe_2O_3: NiO ratio

Okkyun Seo[1,2], Akhil Tayal[1], Jaemyung Kim[1], Chulho Song[1], Yanna Chen[1,2], Satoshi Hiroi[1], Yoshio Katsuya[1], Toshiaki Ina[3], Osami Sakata[1,2,4,*], Yuki Ikeya[4], Shiori Takano[4], Akifumi Matsuda[4,*], and Mamoru Yoshimoto[4]

[1]*Synchrotron X-ray Group, Research Network and Facility Services Division, National Institute for Materials Science (NIMS), Kouto, Sayo, Hyogo 679-5148, Japan*

[2]*Synchrotron X-ray Station at SPring-8, Research Center for Advanced Measurement and Characterization, NIMS, Kouto, Sayo, Hyogo 679-5148, Japan*

[3]*Research & Utilization Division, Japan Synchrotron Radiation Research Institute (JASRI), Kouto, Sayo, Hyogo 679-5148, Japan*

[4]*Department of Materials Science and Engineering, School of Materials and Chemical Technology, Tokyo Institute of Technology, Nagatsuta, Midori-Ku, Yokohama, Kanagawa, 226-8502, Japan*

Corresponding author. E-mail: SAKATA.Osami@nims.go.jp , matsuda.a.aa@m.titech.ac.jp

Surface morphologies of substrate and roughness of the thin films

Figures S1 (a) shows the atomic force microscopy (AFM) image of the sapphire substrate using the tapping mode. The periodic arrays on the sapphire substrate were formed. Figure S1 (b) shows the root mean square roughness of the thin films as a function of Fe_2O_3 content.

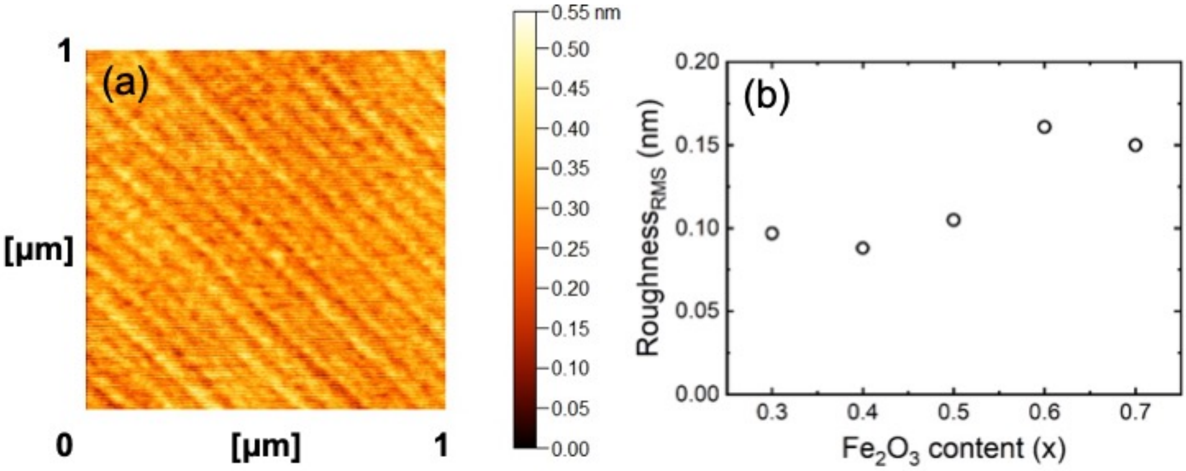

Fig. S1. (a) AFM image of sapphire substrate with periodic arrays and an ultra smooth surface. (b) Surface roughness of $(Fe_2O_3)_{0.5x}$:$(NiO)_{1-0.5x}$ thin films as a function of Fe_2O_3 content.

High-resolution synchrotron X-ray diffraction results of the thin films

Figures S2 (a) and (b) show the high-resolution synchrotron X-ray diffraction patterns of the $(Fe_2O_3)_{0.5x}$: $(NiO)_{1-0.5x}$ thin films as a function of the Fe_2O_3 content along the out-of-plane and in-plane direction. Figure S2 (c) illustrates the azimuthal scan of the thin film with x = 0.3 along the off-specular 002 reflections. Red dash line plots the azimuthal scan of a sapphire substrate along the off-specular $11\bar{2}3$ reflection.

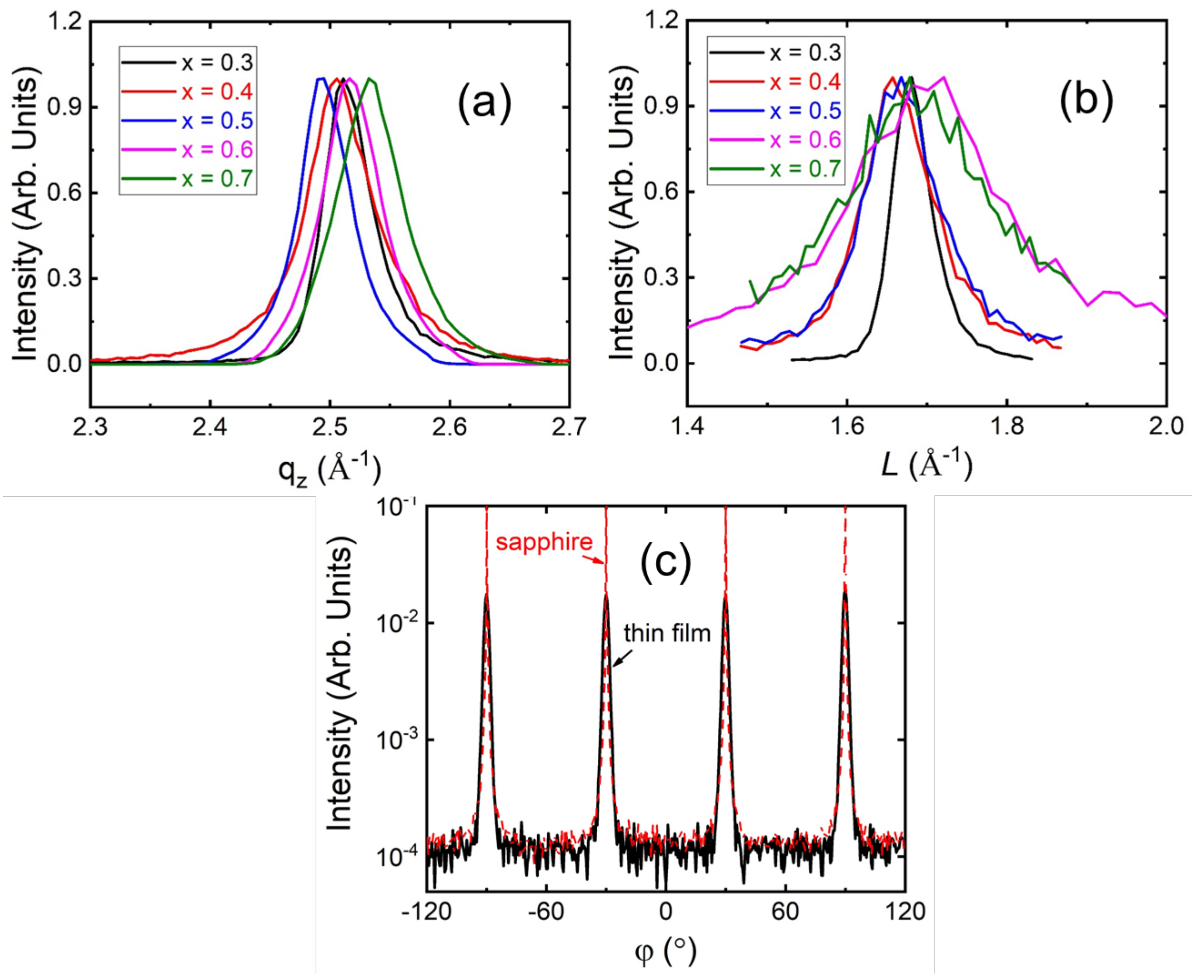

Fig. S2. X-ray diffraction profiles of the thin films along (a) the out-of-plane and (b) in-plane direction as a function of the Fe_2O_3 content. (c) Azimuthal scan profiles of the thin film with x = 0.3 at 002 reflection (black line) and sapphire substrate at $11\bar{2}3$ reflection (red dash line)

Tetragonal distortion of the thin films

Tetragonal distortion of the thin films was evaluated by lattice constants along the out-of-plane and in-plane direction. The relation of tetragonal distortion is $\gamma = \frac{a_{\perp} - a_{\parallel}}{a_{\parallel}}$, where $a_{\perp}$ is the lattice constant along the out-of-plane direction, $a_{\parallel}$ is the lattice constant along the in-plane direction.

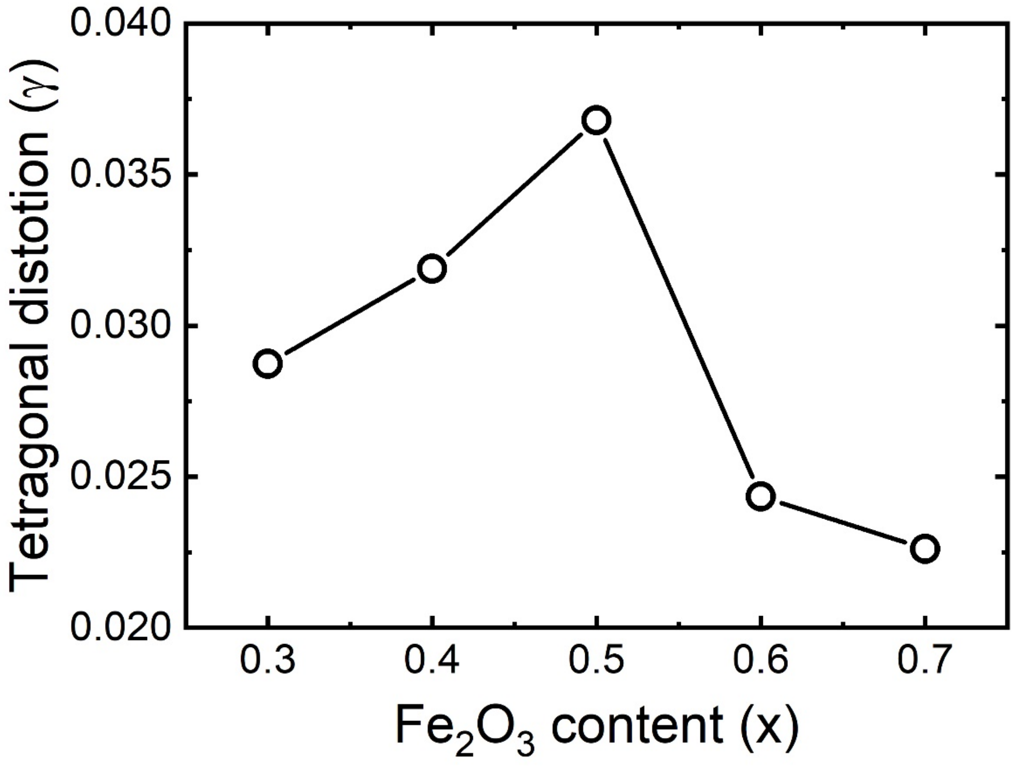

Fig. S3. Tetragonal distortion of the thin films as a function of the Fe_2O_3 content. The relation was estimated from lattice constants along the out-of-plane and in-plane direction.

Pre-edge peak intensity at Fe K-edge region

Figure S4 shows the pre-edge peak intensity at K-edge as a function of Fe_2O_3 content. The pre-edge peak intensity was obtained by subtracting the background from the EXAFS results through an interpolated cubic spline function.

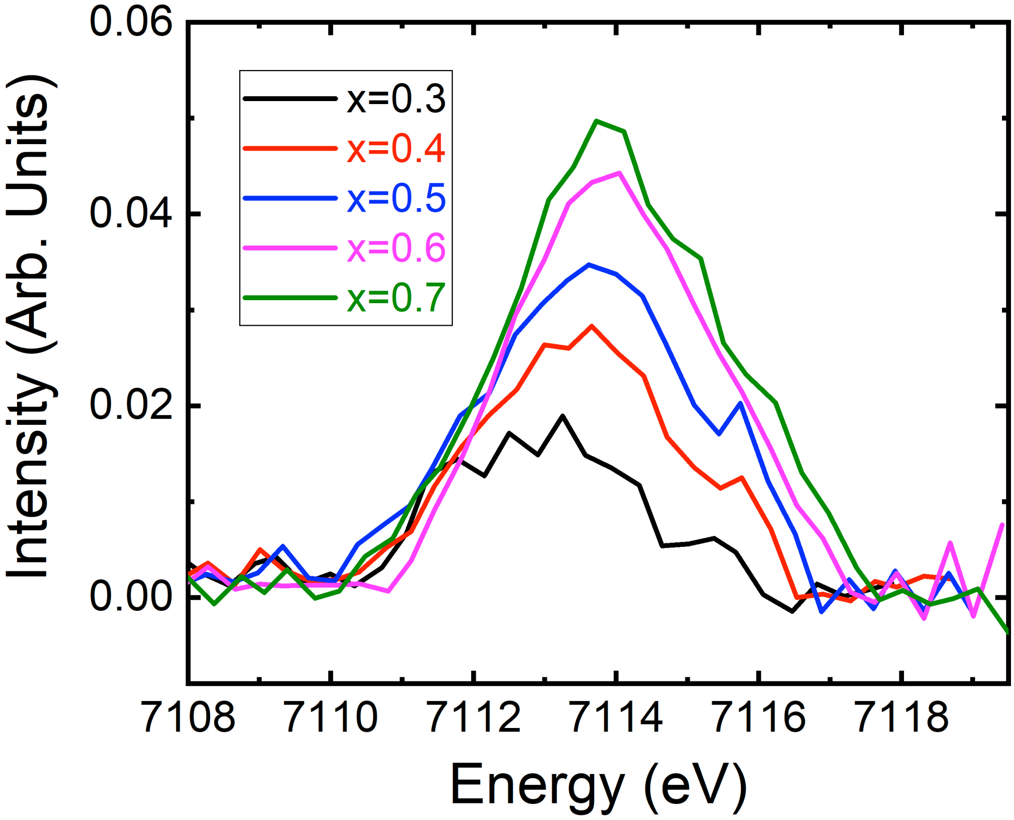

Fig. S4. Variation in the pre-edge peak with Fe_2O_3 content. The pre-edge background region is subtracted using an interpolated cubic spline polynomial function.

Schematic view of the crystal structure of the thin films with Fe_2O_3 content

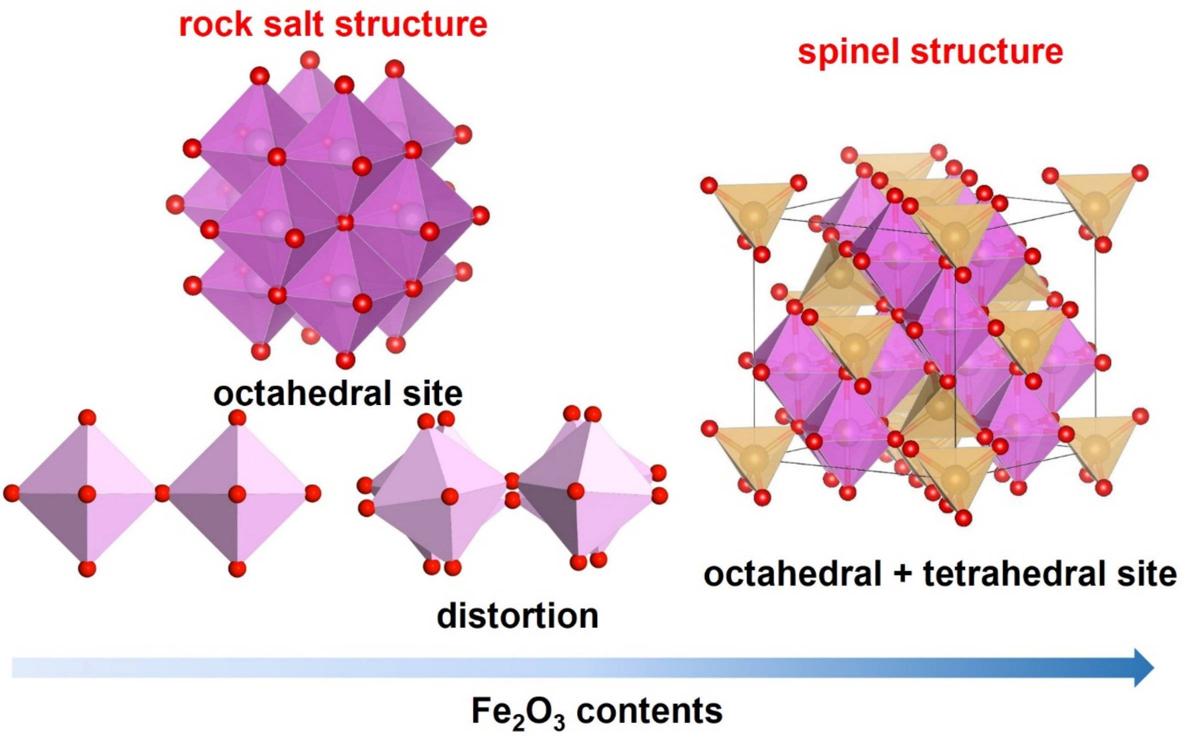

Fig. S5. Schematic illustration of the crystal structure of the thin films with Fe_2O_3 content.

Backward Fourier transform and the logarithm amplitude ratio of Ni-Ni shell

The amplitude loss at higher order shell was correlated with the decrease of the coordination number or the increase of the structural disordering. To evaluate the coordination and structural disordering effects, we were analyzed the EXAFS results by the ratio method, which is able to separate the contribution from the coordination number and the Debye-Waller factor on the amplitude loss. The amplitude of Ni-Ni shell was separated by performing the backward Fourier transform in the region between 2-3.2 Å (Fig. S6). The $\chi(k)$ amplitude falls non-uniformly in the entire k range with much rapid loss occurs in the high k region, indicating the fall in the amplitude have primarily dependence on the increase in the disorder, compared with the coordination number. To separate their contribution, the logarithm amplitude ratio of Ni-Ni shell with the amplitude of Ni-Ni shell against the x = 0.3 was shown in the Fig. S7. The obtained spectra were linearly fitted. A non-linearity in the high k region could arise due to an overlapping neighboring shell.

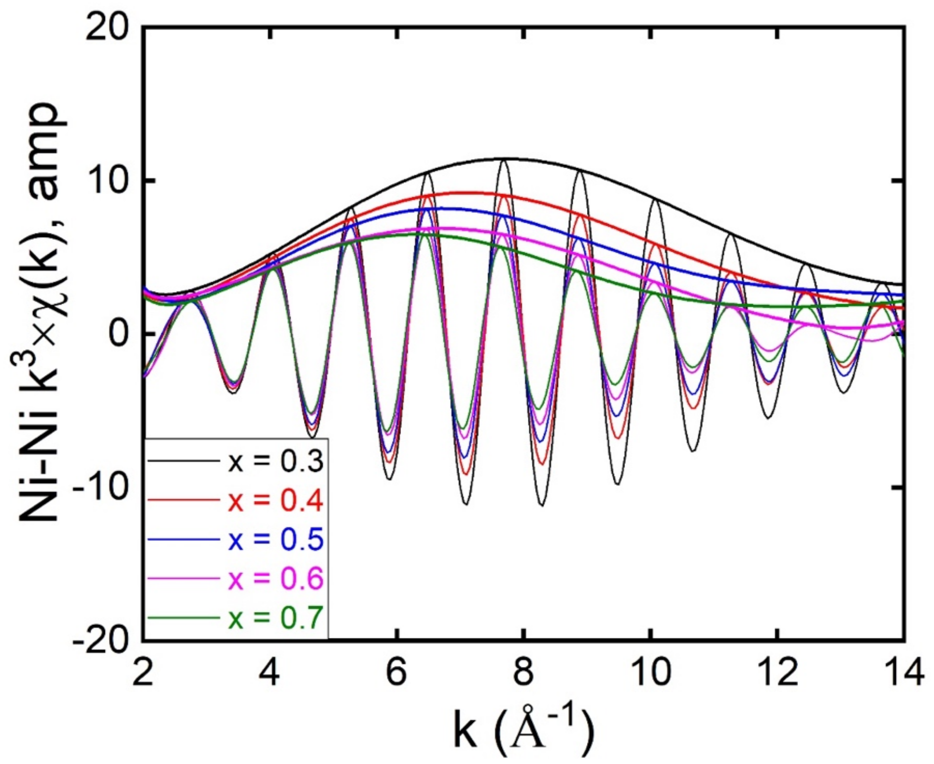

Fig. S6. Ni-Ni $k^3\chi$ spectra of the thin films obtained after performing backward Fourier transform in the region between 2 and 3.1 Å and corresponding amplitudes

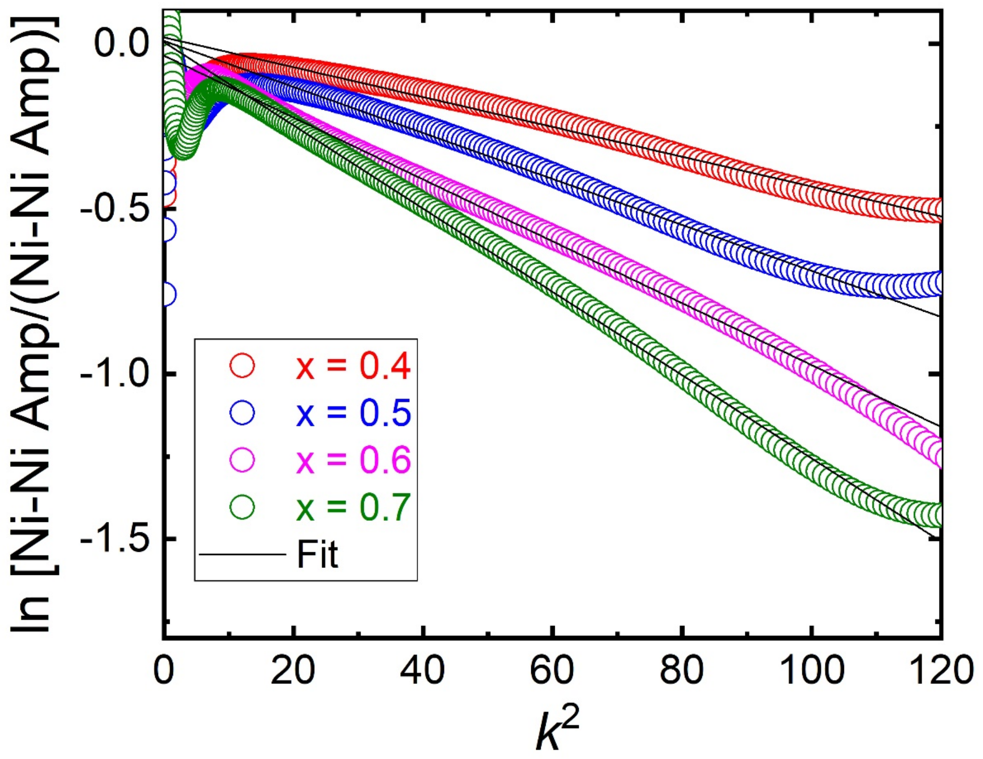

Fig. S7. Logarithms of the ratio of Ni-Ni amplitude with Ni-Ni amplitude of the sample with Fe_2O_3 content at $x = 0.3$ is plotted against k^2 and linear fit to the spectra.

Optical band gap with in-direct transition

The optical band gaps for in-direct transition evaluated using a Tauc's plot using the relation between $(\alpha h\nu)^n$ and $h\nu$ from the absorption edge determined from UV-VIS spectroscopy, where a is the optical absorption coefficient, *h* is Planck's constant, *n* is the photon frequency, and *n* = 0.5 for in-direct transition. The optical band gaps for in-direct transition was 1.27 to 0.78 eV with increasing Fe_2O_3 content.

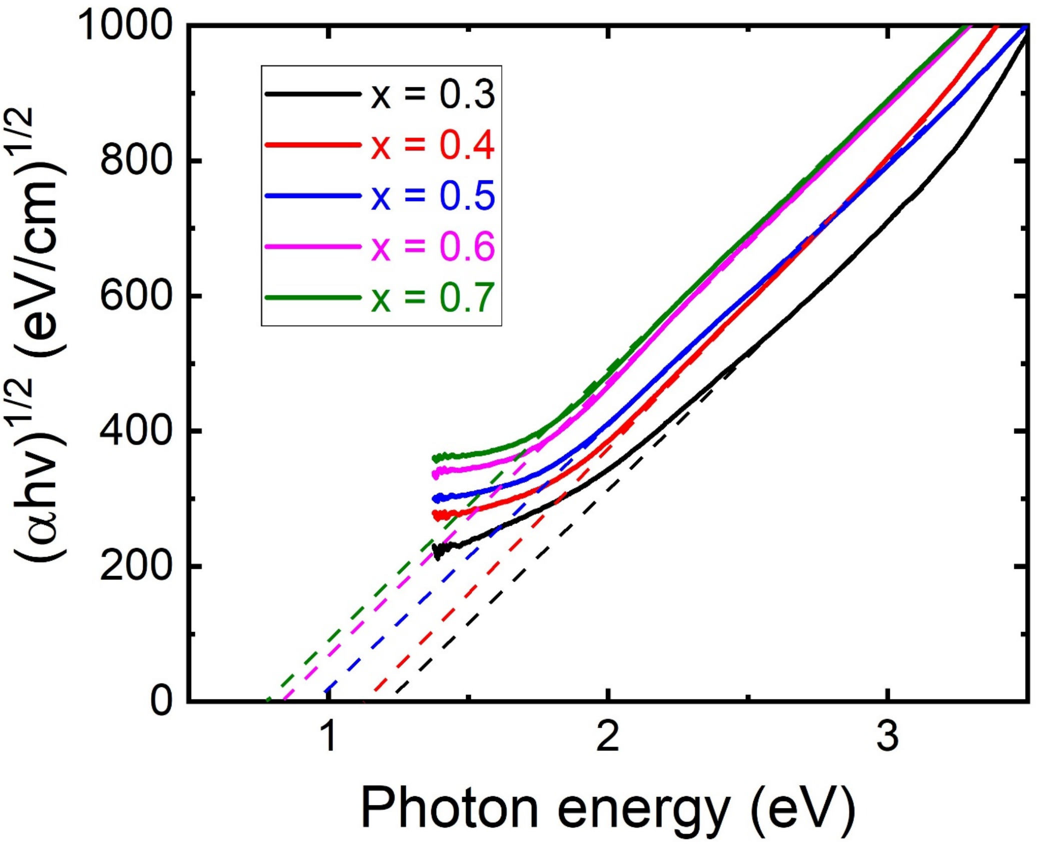

Fig. S8. Tauc's plot of the thin films, with the data obtained from the optical transmittance spectra. The observed optical band gaps of in-direct transition for Fe_2O_3 contents x = 0.3, 0.4, 0.5, 0.6, and 0.7 are 1.27, 1.14, 0.96, 0.84, and 0.78 eV, respectively.
